# Wearable single-lead ECG devices to improve cardiac arrhythmia screening in Ethiopia: Opportunities and challenges

**DOI:** 10.1016/j.hroo.2026.01.002

**Published:** 2026-01-10

**Authors:** Zemichael Getu Alemayehu, Brook Lelisa Sime, Abenezer Shiferaw Keraga, Yonatan Abbawa Zewdie, Henok Wolde Nida

**Affiliations:** Addis Ababa University, College of Health Sciences, School of Medicine, Addis Ababa, Ethiopia

**Keywords:** Arrhythmia, Cardiac arrhythmia screening, Digital health, Ethiopia, Wearable ECG

**Key Findings**

▪ A wearable single-lead electrocardiogram can facilitate viable screening of atrial fibrillation in Ethiopia where there is limited diagnostic infrastructure.
▪ Automated detection is satisfactory, but clinician review significantly enhances accuracy.
▪ Integration with existing health systems is essential to ensure follow-up and clinical impact.
▪ Sustainable implementation in low-resource settings requires capacity building and system-level support.

Cardiac arrhythmia, especially atrial fibrillation (AF), is a major cause of cardiovascular morbidity and mortality worldwide, particularly by raising the risk of heart failure and stroke. In low- and middle-income countries (LMICs) such as Ethiopia, the AF burden is still poorly characterized, in part because of the insufficient diagnostic infrastructure and poor population-level surveillance, and thus prevents the correct estimation of the real prevalence and impact of the disease.^1^

The Heart of Ethiopia: Focus on Atrial Fibrillation (TEFF-AF) study assessed community screening by the use of the KardiaMobile single-lead electrocardiogram (ECG) device in a semirural Ethiopian cohort. Of the 3000 participants, the overall AF prevalence was 0.43%, with an increase to 6.67% in the group of participants aged 70 years and older.^2^ The automated rhythm classification was successful in 78.4% of the original records and 86.7% of repeat records. The high rates of noisy tracings, tachycardia, and ectopy frequently yielded unclassified results, yet manual cardiologist readings did enhance classification accuracy to 97%, which underscores the need for expert control over wearable ECG technology implementation in low-resource settings.^3^ The workflow and impact of wearable single-lead ECG screening in Ethiopia are presented in Figure 1.

**Figure 1:**
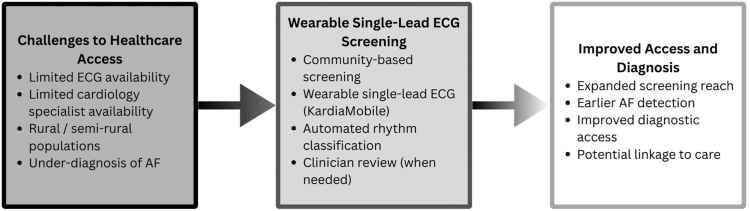
Wearable single-lead ECG screening to improve access and diagnosis of atrial fibrillation in Ethiopia. AF = atrial fibrillation; ECG = electrocardiogram.

Wearable single-lead ECG devices provide an alternative, portable, point-of-care monitoring that could partially address geographic and specialist shortages and could allow earlier arrhythmia detection in resource-constrained environments.^1^^,^^4^ In Ethiopia, where the capacity of cardiac care is limited and access to diagnostic devices and trained staff is restricted, these devices would enable the expansion of the screening range, especially in semirural and rural regions, as evidenced by the TEFF-AF community screening project with a single-lead ECG device.^2^

The adoption of wearable single-lead ECG screening in Ethiopia and other low-resource environments might face the issues that are widely reported in cardiovascular and digital health care delivery to LMICs. These are a limited capacity of health systems, a lack of sufficiently trained staff to interpret ECGs and manage arrhythmias, and the fact that a reliable system is required to enhance follow-up services after the detection of AF. A lack of integration of screening programs in current health services and evident management channels can decrease the clinical effectiveness of screening programs even with successful cardiac arrhythmia detection.^1^^,^^2^^,^^4^

Possible interventions to maximize the benefits of wearable ECG screening are the incorporation of these technologies into the current community-based health interventions and specific capacity-building activities to improve ECG interpretation and arrhythmia management at the primary level of care. Community engagement and integration of screening into routine care pathways could enhance uptake, enable proper follow-up, and assist in translating data generated by devices into clinically relevant decisions.^2^^,^^4^ Enhancing local clinical abilities and alignment with the current health systems frameworks will play a vital role in attaining sustainable, population-level advantages of wearable cardiac monitoring technologies.

In conclusion, wearable single-lead ECG devices provide a feasible, scalable solution for cardiac arrhythmias early detection in Ethiopia. The TEFF-AF study evidence illustrates the feasibility, but clinician participation and system-level support are necessary. To ensure the greatest cardiovascular benefit at the population level in Ethiopia and similar LMIC contexts, performance optimization by training, sociotechnical barrier management, and integration of wearables with overall care pathways will be essential.

## Disclosures

The authors have no conflicts of interest to disclose.
